# Supplementary figures and images for: Development of hidden Markov modeling method for molecular orientations and structure estimation from high-speed atomic force microscopy time-series images
Source: PLoS Comput Biol. 2022 Dec 29;18(12):e1010384. doi: 10.1371/journal.pcbi.1010384 (PMC9833559; doi:10.1371/journal.pcbi.1010384)

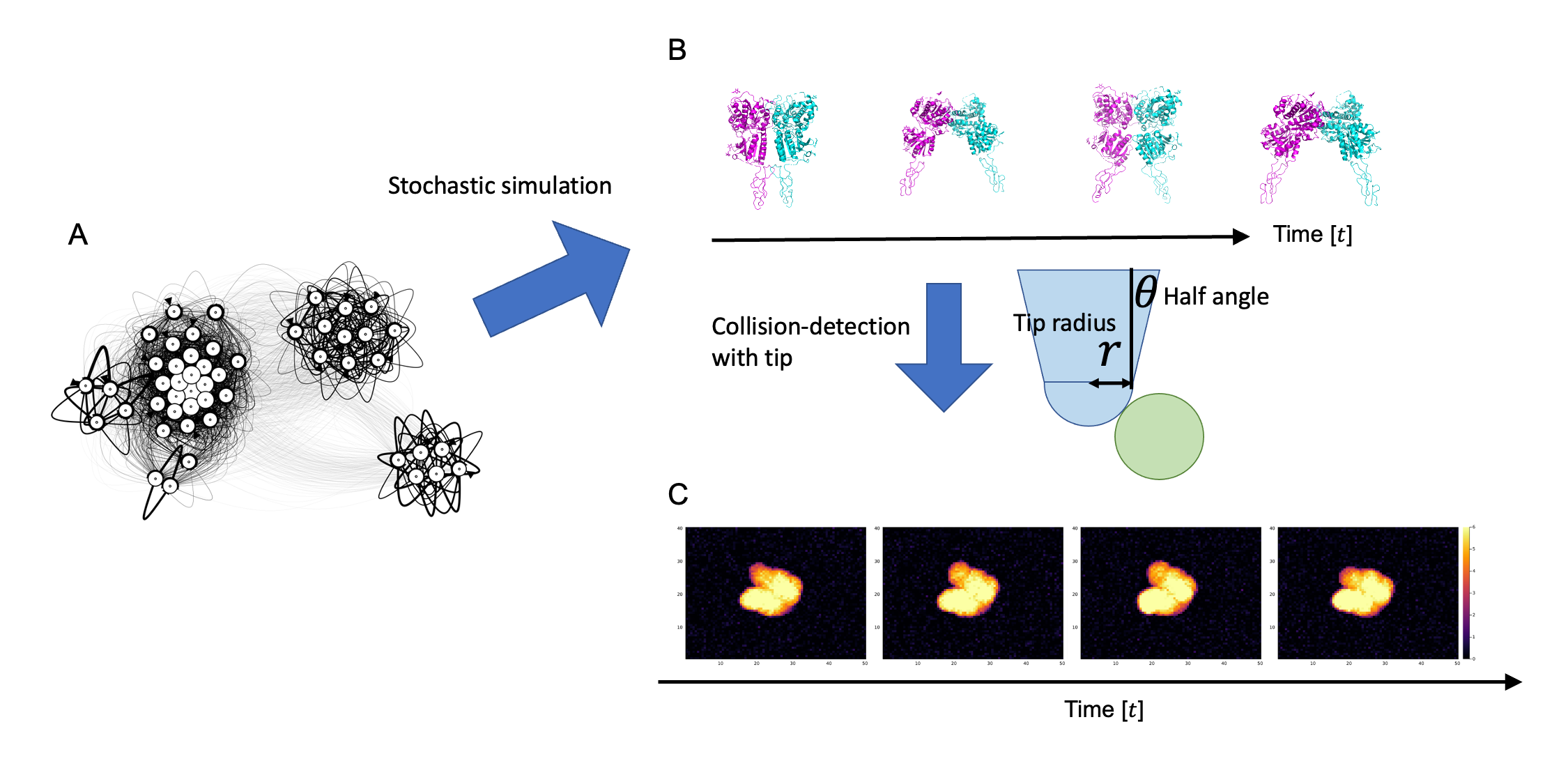

Supplement: S1 Fig — (A) Markov state model constructed from coarse-grained molecular dynamics simulation of the taste receptor type 1. (B) A series of structures were generated by stochastic simulation of the MSM and by drawing the centroid structures of the MSM states. (C) After rotating the structures to a specific orientation, the series of AFM image were generated by the collision-detection with the tip. Finally, spatially independent Gaussian noise were added to pixels. (TIF) [file pcbi.1010384.s001.tif]

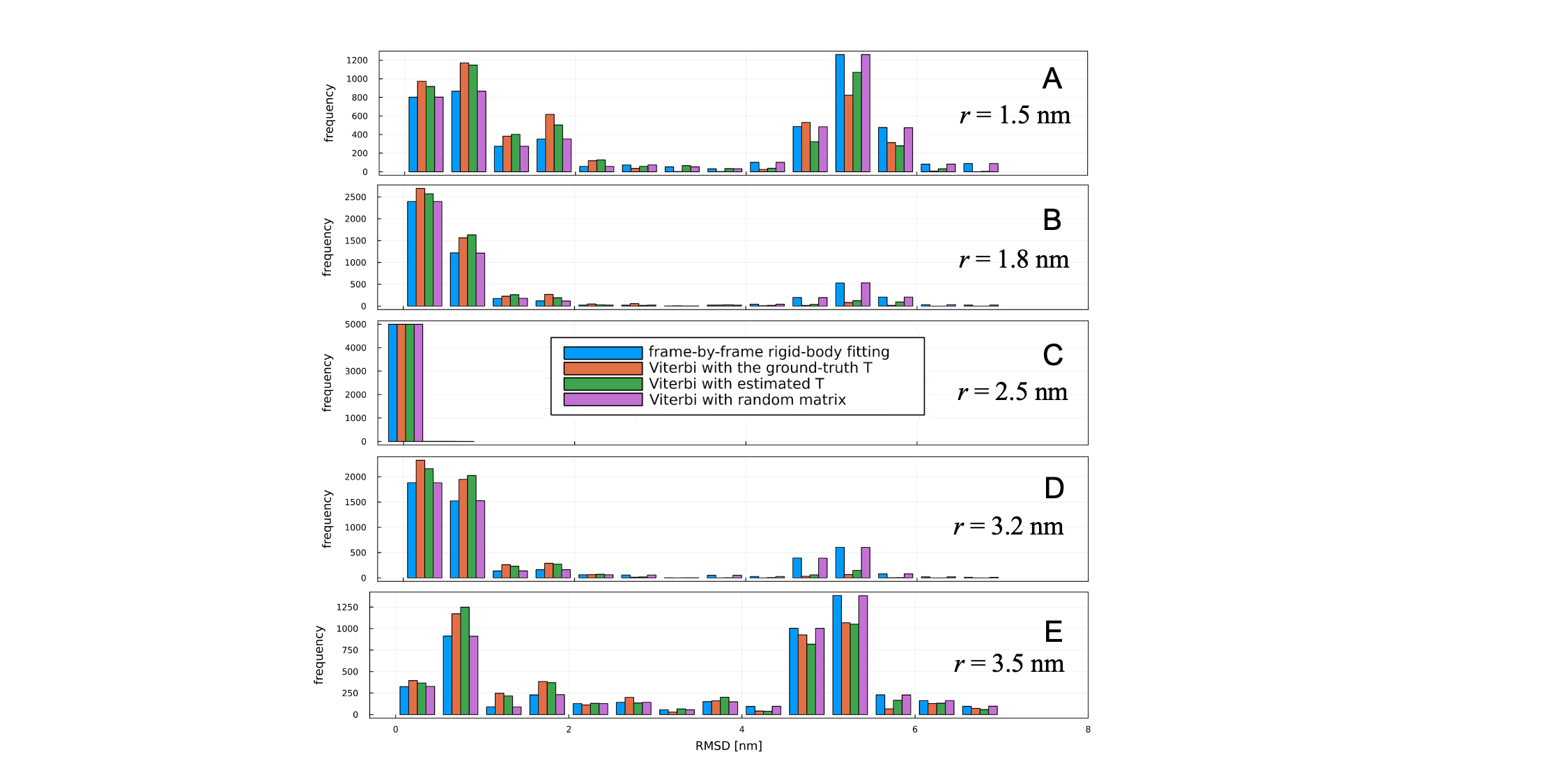

Supplement: S2 Fig — Histograms of root-mean square deviations (RMSDs) of estimated structures from the ground-truth structures in twin experiments. Note that the RMSDs were computed without structural alignment. Structural estimations were performed with various conditions: with different algorithms (the frame-by-frame rigid-body fitting, the Viterbi algorithm using the ground-truth transition probabilities, estimated probabilities by the Baum-Welch algorithm, and a random matrix), and different tip radii (1.5 nm, 1.8 nm, 2.5 nm that is the ground-truth, 3.2 nm, 3.5 nm). (TIF) [file pcbi.1010384.s002.tif]

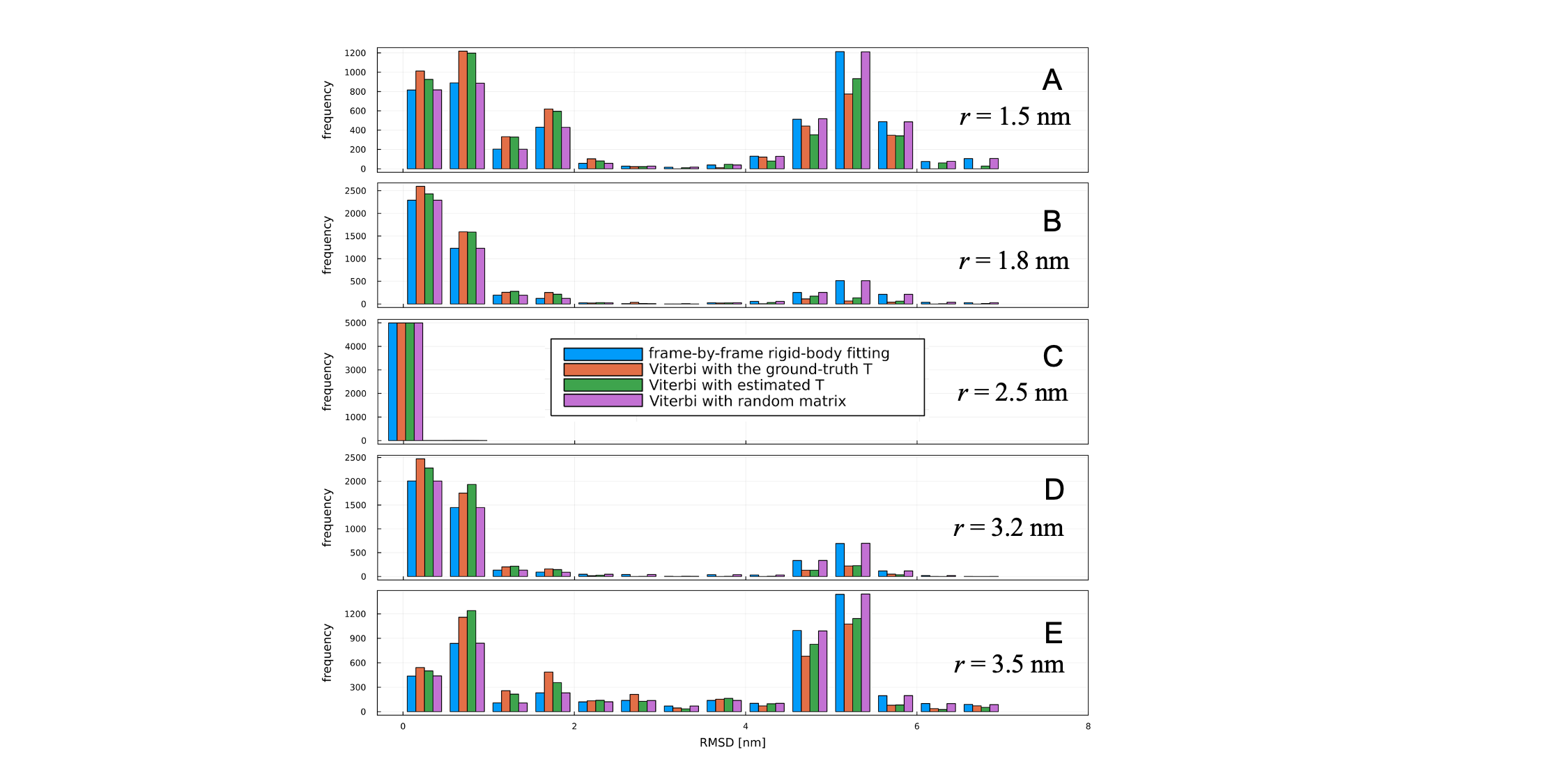

Supplement: S3 Fig — Histograms of root-mean square deviations (RMSDs) of estimated structures from the ground-truth structures in twin experiments. Note that the RMSDs were computed without structural alignment. Structural estimations were performed with various conditions: with different algorithms (the frame-by-frame rigid-body fitting, the Viterbi algorithm using the ground-truth transition probabilities, estimated probabilities by the Baum-Welch algorithm, and a random matrix), and different tip radii (1.5 nm, 1.8 nm, 2.5 nm that is the ground-truth, 3.2 nm, 3.5 nm). (TIF) [file pcbi.1010384.s003.tif]

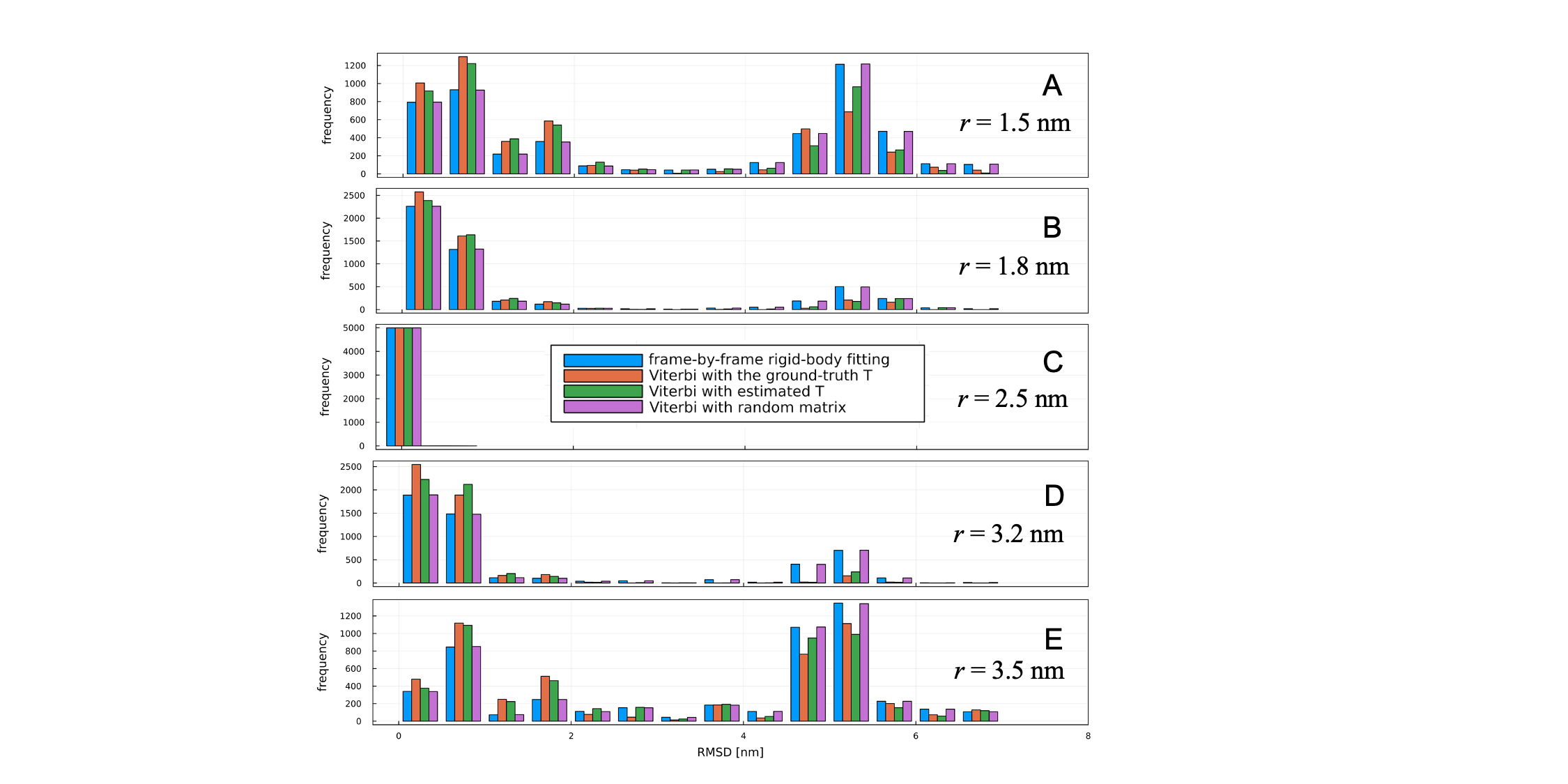

Supplement: S4 Fig — Histograms of root-mean square deviations (RMSDs) of estimated structures from the ground-truth structures in twin experiments. Note that the RMSDs were computed without structural alignment. Structural estimations were performed with various conditions: with different algorithms (the frame-by-frame rigid-body fitting, the Viterbi algorithm using the ground-truth transition probabilities, estimated probabilities by the Baum-Welch algorithm, and a random matrix), and different tip radii (1.5 nm, 1.8 nm, 2.5 nm that is the ground-truth, 3.2 nm, 3.5 nm). (TIF) [file pcbi.1010384.s004.tif]

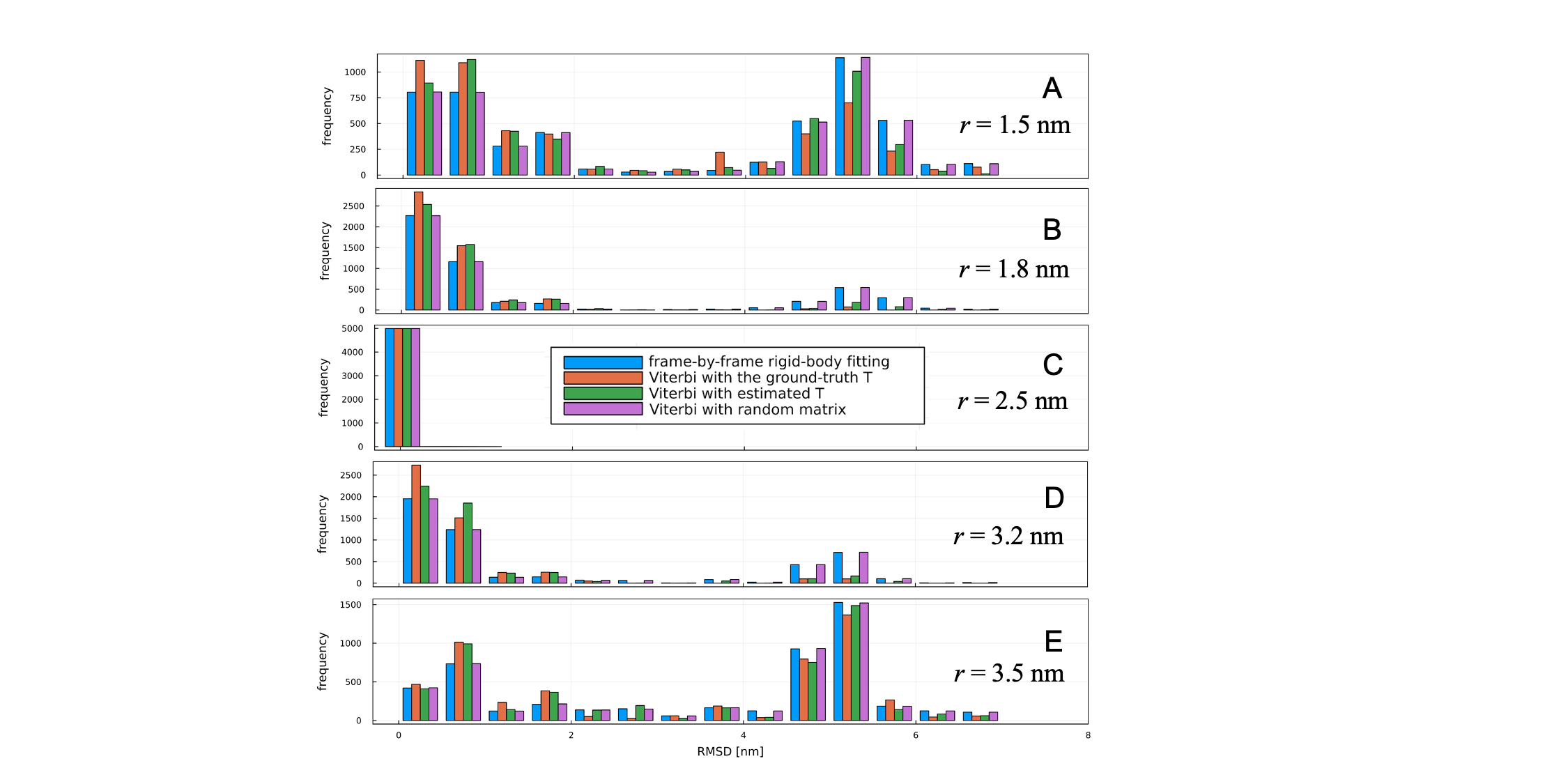

Supplement: S5 Fig — Histograms of root-mean square deviations (RMSDs) of estimated structures from the ground-truth structures in twin experiments. Note that the RMSDs were computed without structural alignment. Structural estimations were performed with various conditions: with different algorithms (the frame-by-frame rigid-body fitting, the Viterbi algorithm using the ground-truth transition probabilities, estimated probabilities by the Baum-Welch algorithm, and a random matrix), and different tip radii (1.5 nm, 1.8 nm, 2.5 nm that is the ground-truth, 3.2 nm, 3.5 nm). (TIF) [file pcbi.1010384.s005.tif]

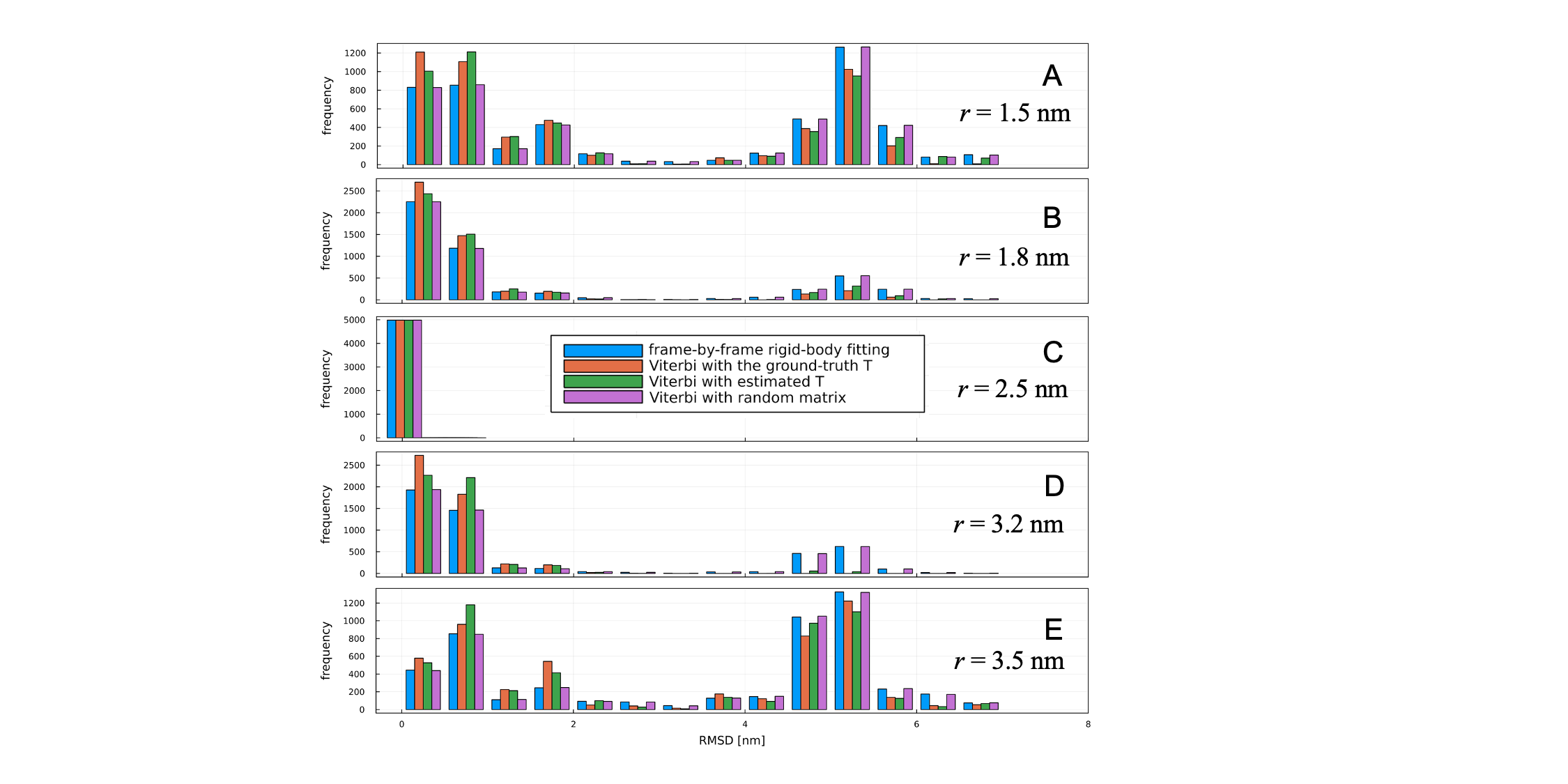

Supplement: S6 Fig — Histograms of root-mean square deviations (RMSDs) of estimated structures from the ground-truth structures in twin experiments. Note that the RMSDs were computed without structural alignment. Structural estimations were performed with various conditions: with different algorithms (the frame-by-frame rigid-body fitting, the Viterbi algorithm using the ground-truth transition probabilities, estimated probabilities by the Baum-Welch algorithm, and a random matrix), and different tip radii (1.5 nm, 1.8 nm, 2.5 nm that is the ground-truth, 3.2 nm, 3.5 nm). (TIF) [file pcbi.1010384.s006.tif]

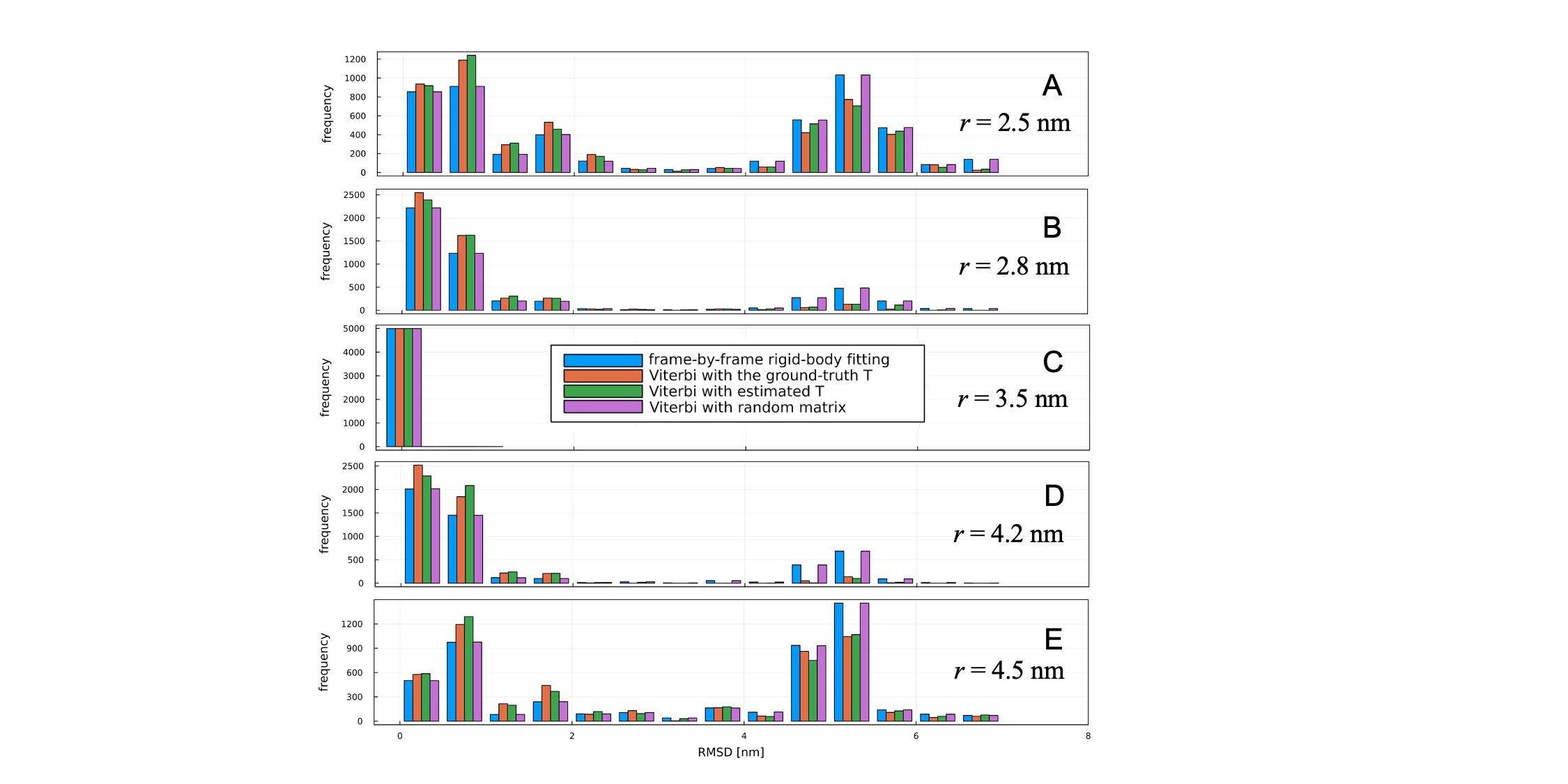

Supplement: S7 Fig — Histograms of root-mean square deviations (RMSDs) of estimated structures from the ground-truth structures in twin experiments. Note that the RMSDs were computed without structural alignment. Structural estimations were performed with various conditions: with different algorithms (the frame-by-frame rigid-body fitting, the Viterbi algorithm using the ground-truth transition probabilities, estimated probabilities by the Baum-Welch algorithm, and a random matrix), and different tip radii (2.5 nm, 2.8 nm, 3.5 nm that is the ground-truth, 4.2 nm, 4.5 nm). (TIF) [file pcbi.1010384.s007.tif]

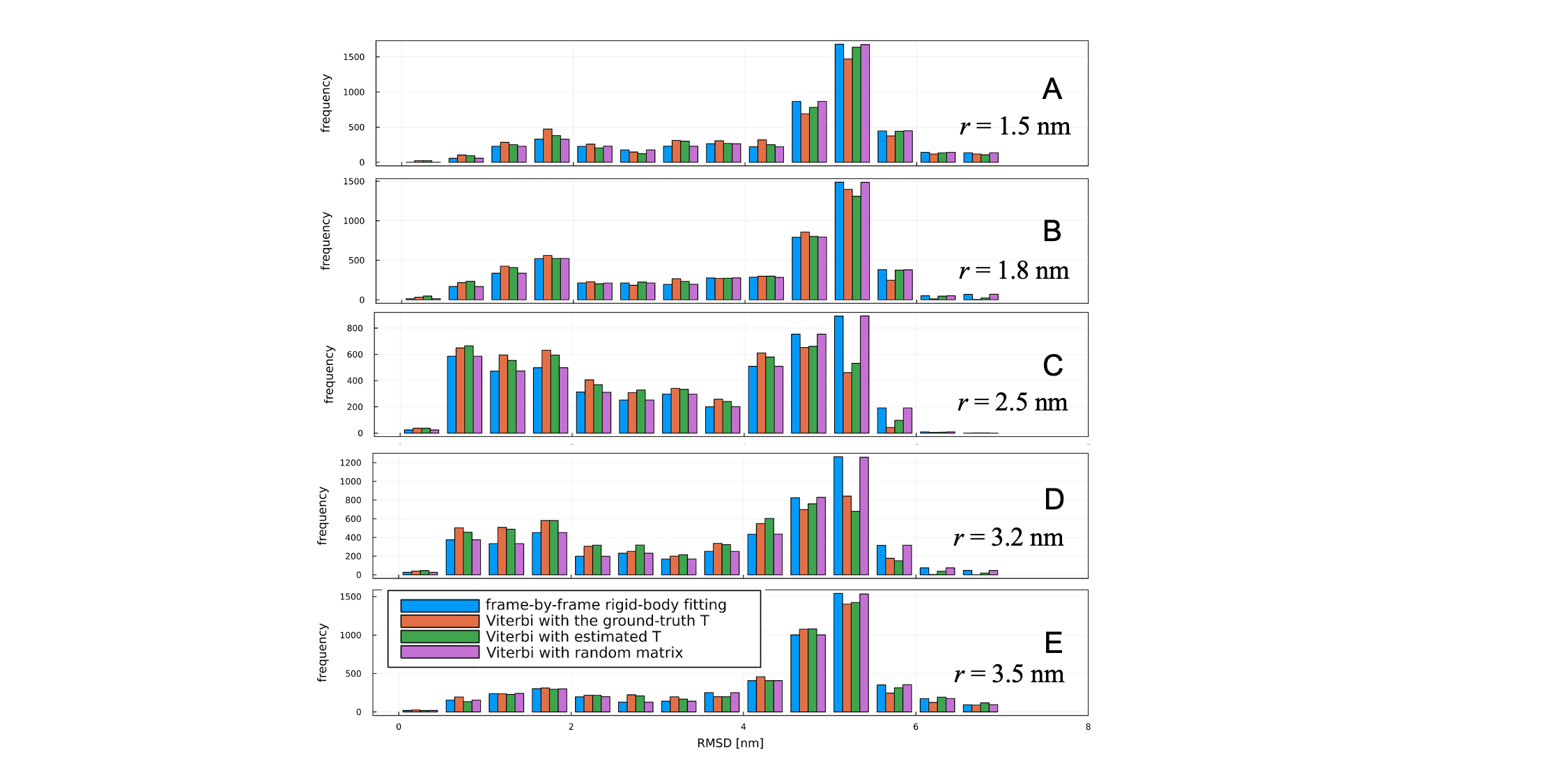

Supplement: S8 Fig — Histograms of root-mean square deviations (RMSDs) of estimated structures from the ground-truth structures in twin experiments. Note that the RMSDs were computed without structural alignment. Structural estimations were performed with various conditions: with different algorithms (the frame-by-frame rigid-body fitting, the Viterbi algorithm using the ground-truth transition probabilities, estimated probabilities by the Baum-Welch algorithm, and a random matrix), and different tip radii (1.5 nm, 1.8 nm, 2.5 nm that is the ground-truth, 3.2 nm, 3.5 nm). (TIF) [file pcbi.1010384.s008.tif]

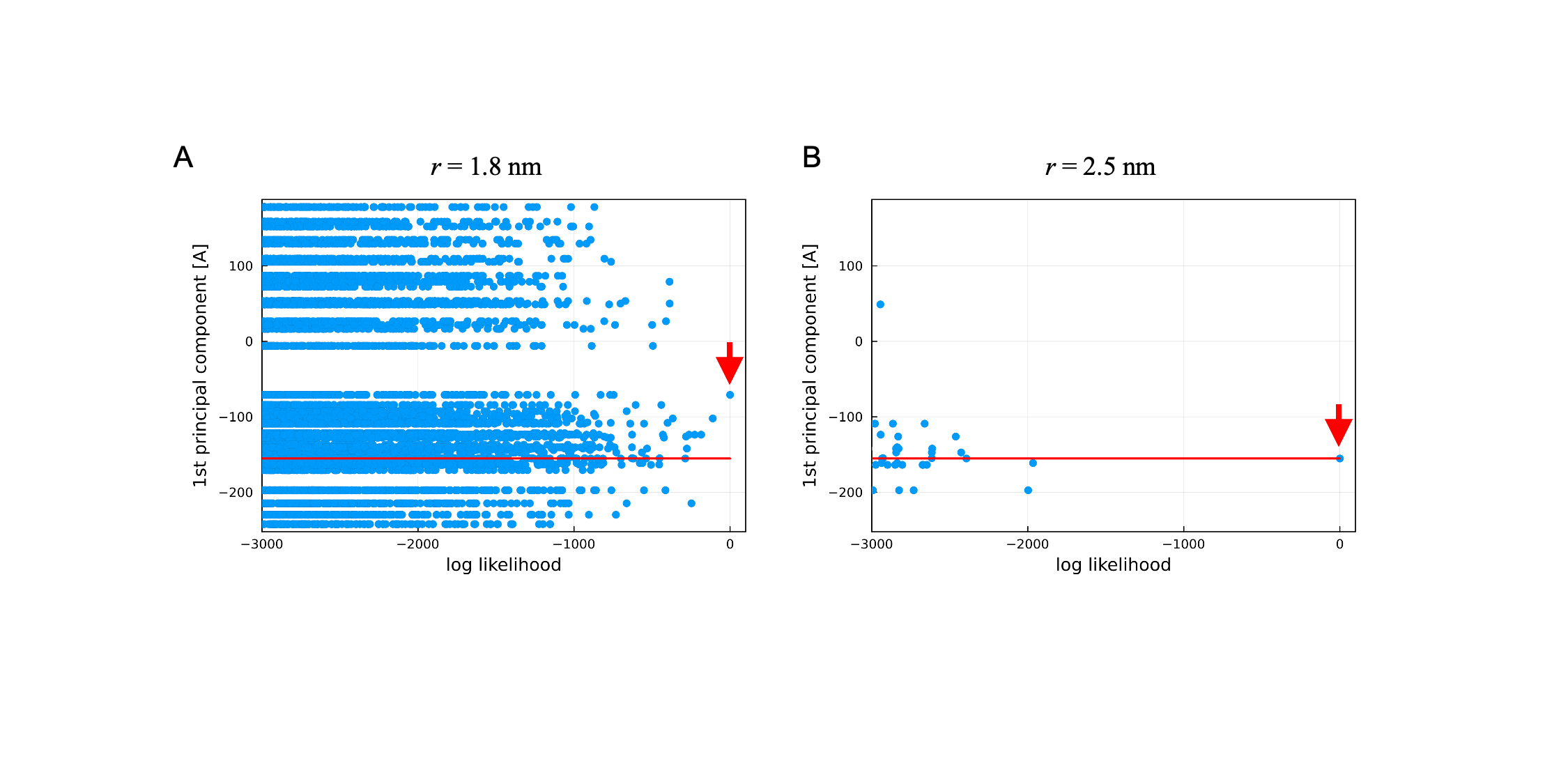

Supplement: S9 Fig — Likelihoods are projected on to the 1st principal components (PC) of the Cartesian coordinates of structures. The maximum likelihoods are indicated by red arrows. The 1st PC of the ground-truth structure is indicated by red solid lines. (A) Likelihoods computed with the tip apex radius of 1.8 nm. (B) 2.5 nm (the ground-truth tip radius). (TIF) [file pcbi.1010384.s009.tif]

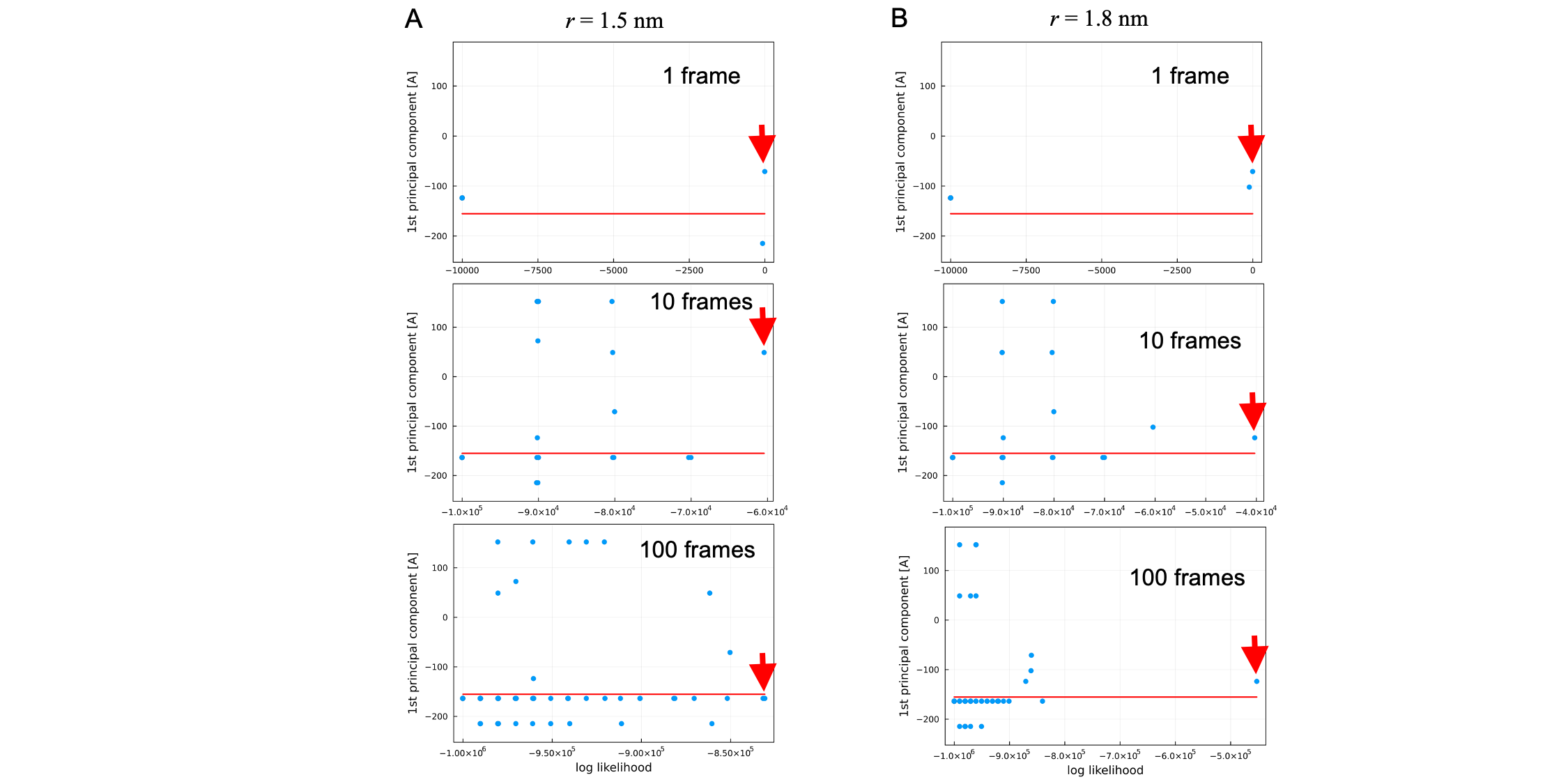

Supplement: S10 Fig — Likelihood values are projected onto the 1st principal component of the structure of the first frame. Note that only the maximum likelihood of each orientation is shown due to computational costs (calculated by the Viterbi algorithm). The maximum likelihoods are indicated by red arrows. The 1st PC of the ground-truth structure is indicated by red solid lines. (A) Likelihoods computed with the tip apex radius of 1.5 nm. (B) 1.8 nm. (TIF) [file pcbi.1010384.s010.tif]

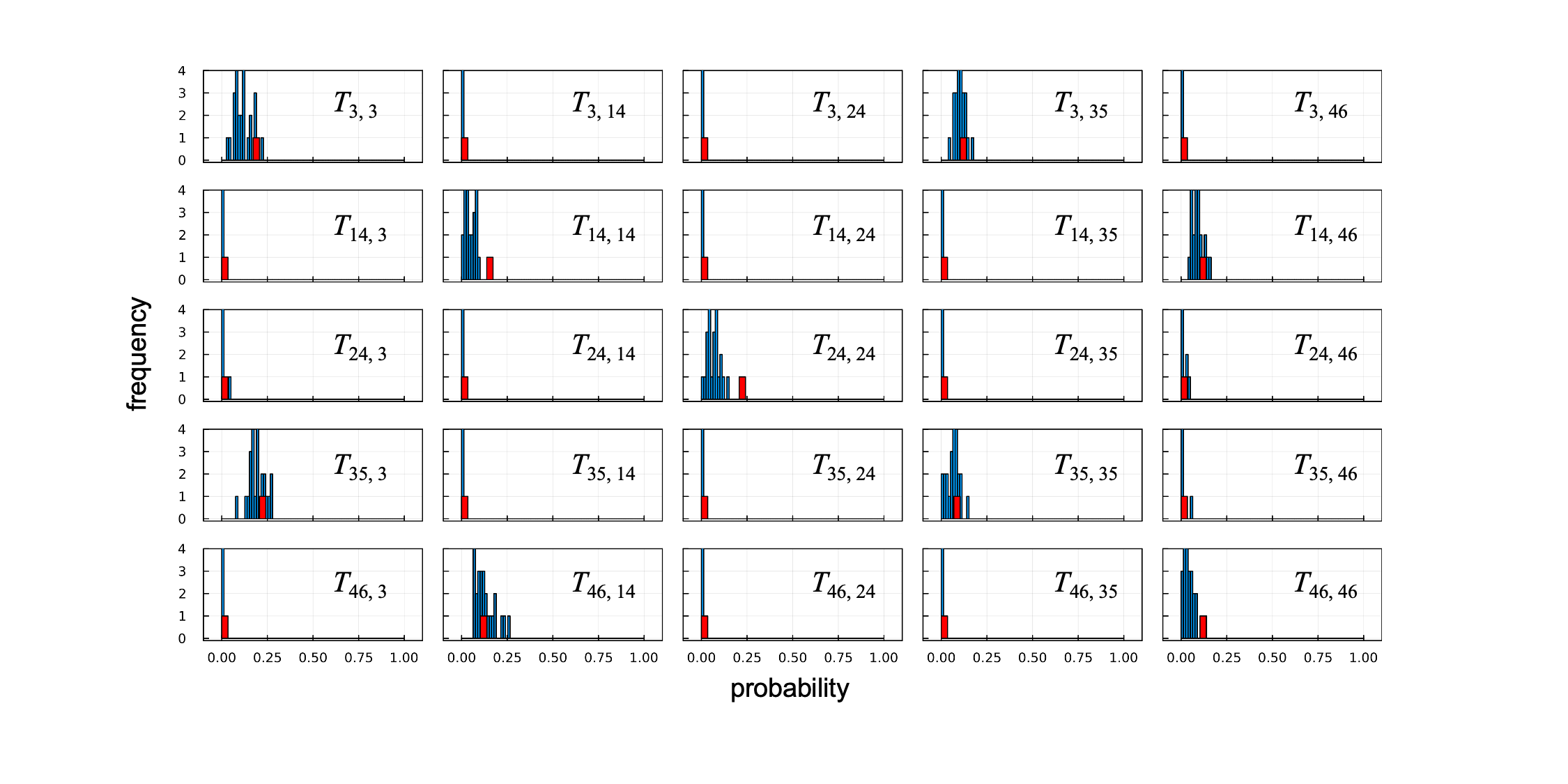

Supplement: S11 Fig — Blue bars indicate 25 estimated transition probabilities from the bootstrap sampling. Red bars indicate the ground-truth transition probabilities. (TIF) [file pcbi.1010384.s011.tif]

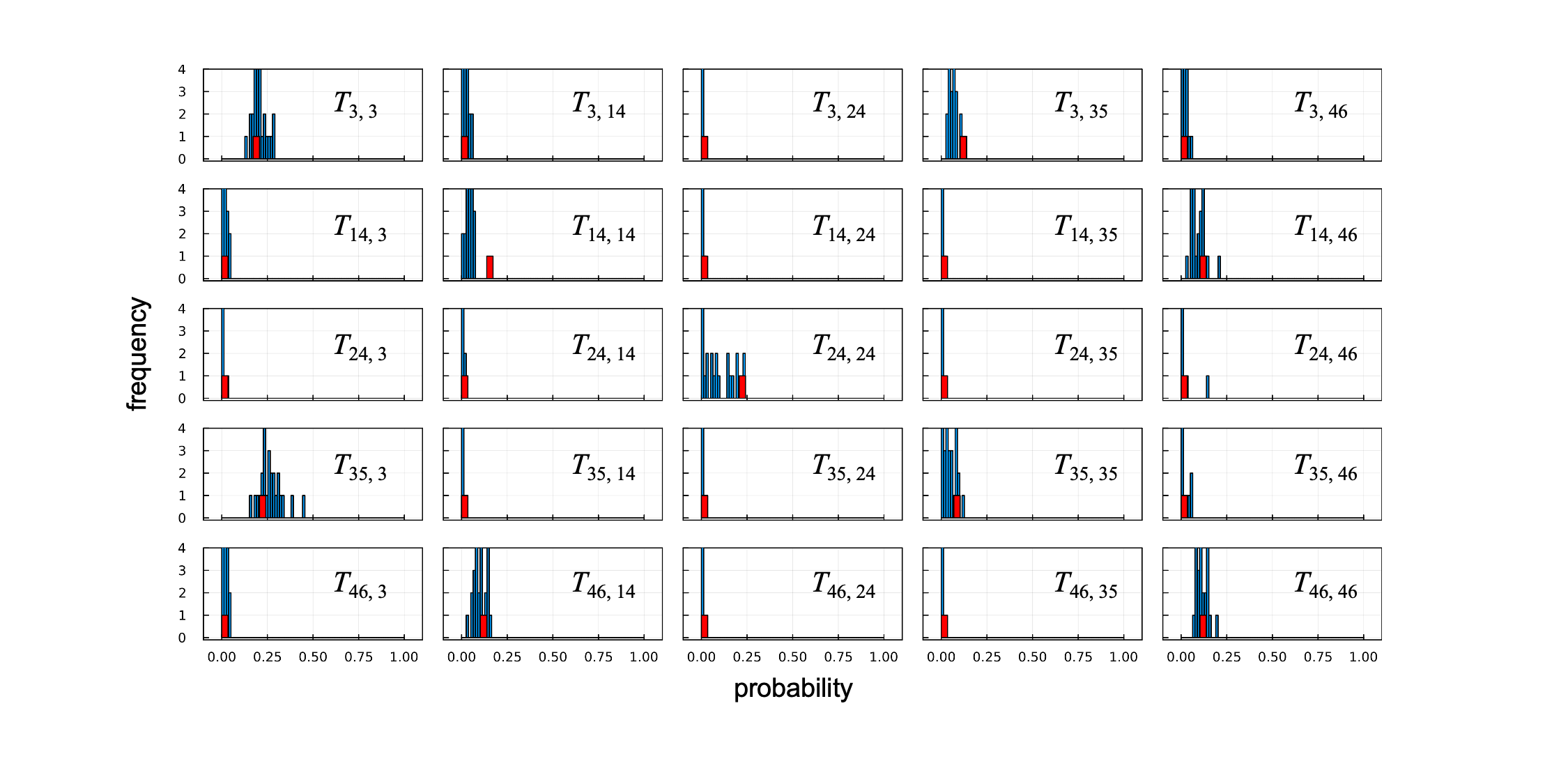

Supplement: S12 Fig — Blue bars indicate 25 estimated transition probabilities from the bootstrap sampling. Red bars indicate the ground-truth transition probabilities. (TIF) [file pcbi.1010384.s012.tif]

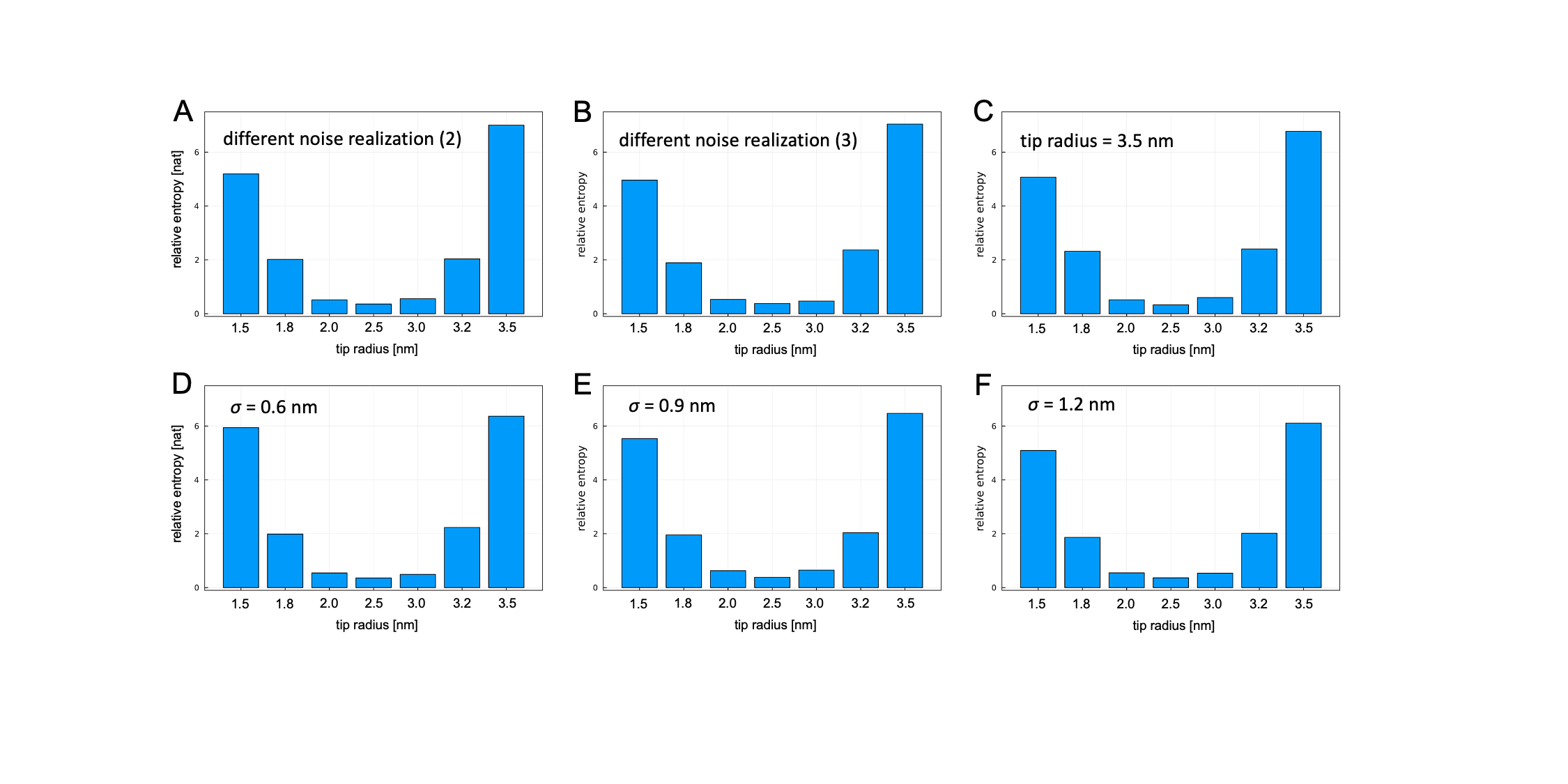

Supplement: S13 Fig — (A) Different pseudo-random number seeds used for Gaussian noise in pseudo-AFM images. (B) Another set of different pseudo-random number seeds for Gaussian noise. (C) Tip radius of 3.5 nm (the ground-truth is 2.5 nm). (D) Standard deviation of 0.6 nm for Gaussian noise in pseudo-AFM images. (E) Standard deviation of 0.9 nm. (F) Standard deviation of 1.2 nm. (TIF) [file pcbi.1010384.s013.tif]
